# Karyotypic complexity and mutated *TP53* are associated with myeloid neoplasms with ring chromosomes

**DOI:** 10.1016/j.htct.2026.108544

**Published:** 2026-10-07

**Authors:** Thaís Gonçalves dos Santos, Mauren Fernanda Moller dos Santos, Renata Kiyomi Kishimoto, Daniela Borri, Caroline Nunes Silveira, Susana Elaine Alves Rosa, Luiz Gustavo Ferreira Cortes, Paulo Campregher, Elvira Deolinda Rodrigues Pereira Velloso

**Affiliations:** a Hospital Israelita Albert Einstein, Sao Paulo, SP, Brazil; b Hospital das Clínicas da Faculdade de Medicina da Universidade de São Paulo, Sao Paulo, SP, Brazil

**Keywords:** Cytogenetics, Ring chromosomes, Myeloid neoplasms, *TP53* gene, Prognosis

## Abstract

**Introduction:**

This study evaluated the prevalence of ring chromosomes, their association with *TP53* alterations, and their prognostic impact in myeloid neoplasms.

**Methods:**

A retrospective analysis of 12,935 onco-hematological karyotypes from 2017–2022 identified 21 adult patients with myeloid neoplasms harboring ring chromosomes. Fluorescence in situ hybridization was used to assess *TP53* deletions, while next-generation sequencing detected *TP53* mutations.

**Results:**

Ring chromosomes were identified in 0.29% of the cases. The median patient age was 69 years and 67% were male. Acute myeloid leukemia and myelodysplastic neoplasms were the most frequent diagnoses. The median number of karyotypic abnormalities was nine (range: 1–28). Karyotypic complexity, *TP53* deletions, mutations, and multi-hit alterations were observed in 81%, 19%, 78%, and 61% of the cases, respectively. ring chromosomes were most frequently derived from chromosomes 6 and 7. Median overall survival was 3.2 months. *TP53* multi-hit alterations (Hazard ratio: 10.63; p-value = 0.001) and the number of karyotypic abnormalities (Hazard ratio: 1.06; p-value = 0.043) were significantly associated with increased mortality. Patients with very complex karyotypes had a 5.53-fold higher risk of death.

**Conclusion:**

Ring chromosomes are rare in myeloid neoplasms and are consistently associated with high karyotypic complexity and *TP53* alterations. In this cohort, both *TP53* alterations and karyotypic complexity emerged as independent prognostic factors and were strongly associated with an extremely poor prognosis.

## Introduction

Ring chromosomes (RCs) are structural chromosomal abnormalities that may be constitutional or acquired in human neoplasms [1,2]. They consist of circular DNA molecules generated after chromosomal damage followed by repair mechanisms [[3], [4], [5]]. According to the International System for Human Cytogenomic Nomenclature (ISCN), a RC is designated by the letter r [6].

The formation of RCs most commonly involves breaks in both chromosomal arms, followed by fusion of the broken ends or by fusion of one broken end with the telomere of the opposite arm, usually resulting in the loss of genetic material [3,7,8]. Less frequently, subtelomeric or telomere-to-telomere fusions may occur, leading to complete rings without apparent loss of genetic material [3,7,8].

Myeloid neoplasms (MNs) comprise a heterogeneous group of hematological disorders, including myelodysplastic syndromes (MDS), acute myeloid leukemia (AML), and myeloproliferative neoplasms (MPNs) [9]. These diseases originate from hematopoietic stem cells of the myeloid lineage, resulting in clonal disorders of myeloid progenitor cells (blasts) [10] Consequently, MDS and AML are characterized by cytopenias affecting one or more hematopoietic lineages, with clinical manifestations commonly related to anemia, thrombocytopenia, and leukopenia, predominantly affecting individuals between 60 and 70 years of age [10,11]. RCs represent a rare and poorly understood cytogenetic abnormality in myeloid neoplasms [2,[11], [12], [13]].

Fluorescence in situ hybridization (FISH), a molecular cytogenetic technique widely used in the diagnostic evaluation of hematological neoplasms, allows the detection of specific chromosomal abnormalities with higher sensitivity than conventional cytogenetic methods [[14], [15], [16]].

The *TP53* gene, located on the short arm of chromosome 17 (17p13.1), encodes the tumor suppressor protein p53 [17,18]. Known as the ‘guardian of the genome,’ p53 plays a key role in maintaining genomic stability [19]. Deletions or mutations in *TP53* allow genetically damaged cells to evade DNA repair and apoptosis, contributing to increased proliferation and survival of neoplastic cells [3].

## Objective

The aim of this study was to evaluate the prognostic significance of RCs in myeloid neoplasms and to investigate their association with *TP53* deletions and mutations, and karyotypic complexity.

## Methods

### *Patients*

This retrospective study involved adult patients (aged 18 or older) diagnosed with myeloid neoplasms and RCs between 2017 and 2022. Cytogenetic and molecular studies were performed at the laboratory of Hospital Israelita Albert Einstein. Treatment decisions were made at the discretion of the attending physicians. All participants signed an informed consent form (ICF) in accordance with the Brazilian Health Ministry Resolution 466/2012.

### *Samples*

Bone marrow samples were collected, fixed in Carnoy's solution and stored at −20 °C. Following incubation at 37 °C for 24 or 48 h, metaphase cells were harvested to obtain cell pellets.

### Fluorescence in situ hybridization (FISH)

The FISH technique was employed following the manufacturer’s instructions. The XL TP53/17cen dual-color probe (MetaSystems Probes, Germany) was utilized to detect deletions at 17p13 involving the *TP53* gene locus. A hundred nuclei were analyzed per slide by two analysts with images being captured using Isis software (MetaSystems, Germany). Reference values for *TP53* gene deletions and chromosome 17 monosomies are below 7.3%.

### *Karyotype and complexity study*

Karyotype data were retrieved from the institutional database, with twenty metaphases analyzed per sample using the Ikaros system (MetaSystems, Germany). Chromosomal abnormalities were classified based on International System for Human Cytogenomic Nomenclature (ISCN) 2020. Karyotypes were categorized as non-complex (<3 abnormalities), complex (CK: 3–4 abnormalities), or very complex (VCK: ≥5 abnormalities) [20].

### *Next-Generation sequencing (NGS) study*

DNA was extracted using the QIAmp DNA Blood Mini kit (Qiagen, Germany) and quantified via the Qubit dsDNA HS Assay kit on a Qubit 2.0 Fluorometer (Life Technologies, USA). Libraries were prepared from 50 nanograms of deoxyribonucleic acid using the TruSight Myeloid sequencing panel and sequenced on the NextSeq platform (Illumina, USA). Data analysis was conducted using an in-house bioinformatics pipeline combined with results from the TruSeq Amplicon software within the BaseSpace platform. Multi-hit *TP53* alterations were defined by: (i) a single mutation with a variant allele frequency (VAF) ≥50%, (ii) the presence of two distinct mutations with a VAF >10%, or (iii) a concurrent deletion and mutation with a VAF >10% [21,22].

### *Data management*

Patient data were managed using the REDCap software platform, which facilitates secure data collection, management, and authenticated access. This platform was also utilized for the generation of informed consent forms and the capture and storage of clinical data.

### Statistical analysis

Demographic, clinical, cytogenetic, and outcome data were entered into REDCap. Quantitative characteristics were described using statistical measures (mean, standard deviation, median, and 25th and 75th percentiles), while qualitative characteristics were presented as absolute and relative frequencies. Survival analysis was performed using Kaplan-Meier curves, with comparisons made using the log-rank test or bivariate Cox regression. The analyses were conducted using IBM-SPSS software and results were tabulated in Microsoft Excel, with the level of significance set at 5%.

## Results

An analysis of 12,935 karyotypes from patients with hematological neoplasms between 2017 and 2022 identified 38 cases with RCs, representing 0.29% of all cases. Of these, eight cases were excluded: five patients could not be located, one patient declined to participate, and three patients had lymphoid neoplasms, resulting in a final cohort of 21 patients.

Demographic characteristics, diagnoses, karyotypic findings, and FISH and NGS results for the *TP53* gene are summarized in Table 1. Among the 21 patients, 66.7% were male, and the median age was 69.0 years (range: 43–89). AML was diagnosed in nine patients (42.9%), while 12 patients (57.1%) had other diagnoses, including MDS (43%), MPNs (4.7%), biphenotypic acute leukemia (BAL; 4.7%), and MDS/MPN overlap syndromes (4.7%). Treatment information was available for six patients (28.6%), all of whom received hypomethylating agents, either alone or in combination with venetoclax (n = 5); four of these patients underwent hematopoietic stem cell transplantation.

**Table 1:** General data.

**Case**	**Sex/Age**	**Disease**	**Karyotype**	***TP53***	***TP53* Mutation**
	**(years)**			**deletion**	**(NM_000546)**
1	M/68	AML	46,X,-Y,?t(1;17)(q41q24),add(3) (p12),del(5)(q22q35),−12,**r(12)** (p13q24.3),+22,+mar[15]	No	Ind.
2	F/79	MDS	46∼47,XX,del(5)(q13q33),del(7) (p21),−18,−20,+22,+22,**+r**, +1∼2mar[cp17]/46,XX[3]	No	c.838A>G p.R280G VAF:17.7%
3	M/67	MDS	46,XY,del(21)(q21)[1]/ 44,sl,del(3)(q24), del(5)(q11.2q33),−17,−18, −20,**+r**[6]/43,sdl,-Y[11]/46,XY[2]	**Yes**	c.515T>A p.V172D VAF:70%
4	M/73	MDS	46,XY,del(9)(q21q22), −21,**+r**[15]/45,idem,−4[5]	No	c.839G>A p.R280K VAF: 47%; c.395del p.K132fs VAF: 51.1%
5	M/84	AML	56,XY,+Y,add(7)(q11.2),+8,+8, +9,+10,+11,add(11)(p11.2), +14,idic(19)(q13.1),+21, +22,+r[9]/57,XY,del(2)(q33), +8,+8,+9,+10,+11,add(11) (p11.2),+14,+18,+19,+21, +22,**+r**[7]/46,XY,del(1)(p36.1), add(7)(p15),−15,+mar[3]/46,XY[1]	No	Ind.
6	M/58	AML	45,XX,der(1)t(1;11)(p34;q13), del(6)(q21q23),del(7) (q31q33–35),−13,−16,−16, add(17)(p11.3),−18,add(18) (p11.2),+22,+mar,**+r**[cp7]/ 45,XX,−12,−16,−16, der(17)t(12;17)(q13;q23), +2mar[4]//46,XY[9]	No	Non-mutated
7	M/56	AML	43∼45,XY,der(3)t(3;12) (q27;q13)ins(3;?)(q21;?),−5,−6, −7,+8,add(11)(q25),−13,del(16) (p11.2),−17,i(17)(q10), add(20)(q11.2),+r,+3∼4mar[cp8]/41,XY,−3,−5,−7,add(11)(p15), +der(11)del(11)(p12)add(11) (q25),add(12)(p12),−13,−16,−17, i(17)(q10),−18,add(19)(p13), add(20)(11.2),−21,−22, **+r**,+2mar[5]/46,XY[7]	No	c.400T>C p.F134L VAF: 66.9%
8	M/70	BAL	40,XX,add(1)(p36.1),−3,−4,del(6) (q21q23),−7,−9,add(9)(q34),−11, del(12)(q12q21),−16,der(16) t(11;16)(q13;q24),−17,−18, add(20)(q13.3),add(22)(q13),**+r**,+mar[18]/46,XX[2]	**Yes**	c.817C>T p.R273C VAF: 84.3%
9	M/69	MDS/MPN	46,XY,**r(7)**[20]	No	Ind.
10	M/53	MPN	46,XY,del(5)(q22q35)[5]/ 60,XYY,−3,−4,−5,del(5)(q22q35),−7,−9,−10,−11,−12,−1 ,−15, ?add(16)(q12–13),−17,−17,−18,−19,add(19)(p13),−21,**+r**, 5∼7mar[cp13]/46,XY[2]	No	c.733G>T p.G245C VAF: 29.9%
11	M/57	AML	44∼46,XY,?dup(1)(p22p36.1),−3,−5,−7,+8, add(11)(p15),−15,−15, **+r**,+2∼4mar[cp17]/46,XY [3]	No	c.488A>G p.Y163C VAF: 36.6%; c.379T>C p.S127P VAF: 37.1%
12	M/69	MDS	47,XY,del(5)(q?22q?33),**r(6)** (p25q13),del(7)(p13), +19[7]/46,XY[2]	No	c.912delT p.K305fs VAF: 27.3%; c.375G>A p.T125T VAF: 27.4%
13	M/76	MDS	47∼49,XY,add(5)(q13),−7, add(11)(p11.2),dic(11;16) (p11.2;p13.1),−16,**r(19)** (p13q13.3),3∼5mar[cp18]/ 46,XY[2]	No	c.578A>G p.H193R VAF: 68.8%
14	M/64	AML	46,XY,add(1)(p34),add(5) (q11.2),+6,−8,−9,del(9)(p21),i(17) (q10),der(22)t(8;22)(?;q13) hsr(8)(q21.3),+mar,20∼40dmin [16]/46,idem,−8,**+r**[4]	**Yes**	c.461delG p.G154fs VAF: 93.3%
15	M/69	MDS	46,X,dic(Y;17)(q12;p11.2), **r(2)**(p23q31),add(5)(q11.2)[3]/ 46,XY[17]	No	c.733G>A p.G245S VAF: 8.2%
16	F/43	AML	46,XX,−5,**r(6)**(p25q25),+8,−17,+mar[6]/46,idem, del(4)(q28),add(7)(p12)[4]/ 46,XX[10]	No	c.524G>A p.R175H VAF: 32.1%; c.919+15C>T VAF: 6.7% (VUS)
17	F/62	AML	46,XX,inv(3)(q21q26.2), **r(7)** (q11q31)[19]/46,XX[1]	No	Non-mutated
18	M/57	MDS	47,XY,+8,**r(20)**(p12q11.2)[20]	No	Non-mutated
19	F/89	MDS	45∼50,XX,−4,del(5)(q12q34),add(7)(q11.2),der(7;19) (q10;q10),+8,del(9)(p13),−13, add(16)(p13.3),del(18)(q12),−19,**r(22)**(p11.2q13),+21, 1∼3mar[cp19]/46,XX[1]	No	c.824G>A p.C275Y VAF: 25%; c.548C>A p.S183* VAF: 31.1%
20	F/73	AML	46,XX,**r(7)**[6]/46,XX[5]	No	Non-mutated
21	M/79	MDS	46∼48,XY,t(1;4)(q32;q12),del(5)(q21q34),del(7) (q11.2q36),+8,+8,+8,+11, dic(15;17)(p11.2;p11.2),−16, **r(18)**(p11.2q22),−20[cp17]/ 46,XY[3]	**Yes**	c.743G>A p.R248Q VAF: 34%

M: Male; F: Female; AML: Acute Myeloid Leukemia; MDS: Myelodysplastic Neoplasms; BAL: Biphenotypic Acute Leukemia; MDS/MPN: Myelodysplastic/Myeloproliferative Neoplasms; MPN: Myeloproliferative Neoplasms; Ind: Indeterminate; ISCN nomenclature: t (translocation), add (additional material), del (deletion), r (ring), mar (marker chromosome), idic (isodicentric), der (derivative chromosome), ins (insertion), i (isochromosome), dup (duplication), dic (dicentric), hsr (homogeneously staining region), dmin (double minutes), inv (inversion), + (gain), - (loss).

The mean and median numbers of karyotypic abnormalities were 11.0 (SD = 8.0) and 9.0 (interquartile range: 4.0–15.5), respectively. Karyotypes were classified as non-complex in 19% (n = 4), CK in 14.3% (n = 3), and VCK in 66.7% (n = 14) of the cases.

The origin of RCs could not be determined in ten cases (47.5%). Among the cases with identifiable origins, RCs most frequently originated from chromosome 7 (n = 3; 14.3%) and chromosome 6 (n = 2; 9.5%). Single instances (4.8% each) were observed involving chromosomes 2, 12, 18, 19, 20, and 22 (Figure 1).

**Figure 1:**
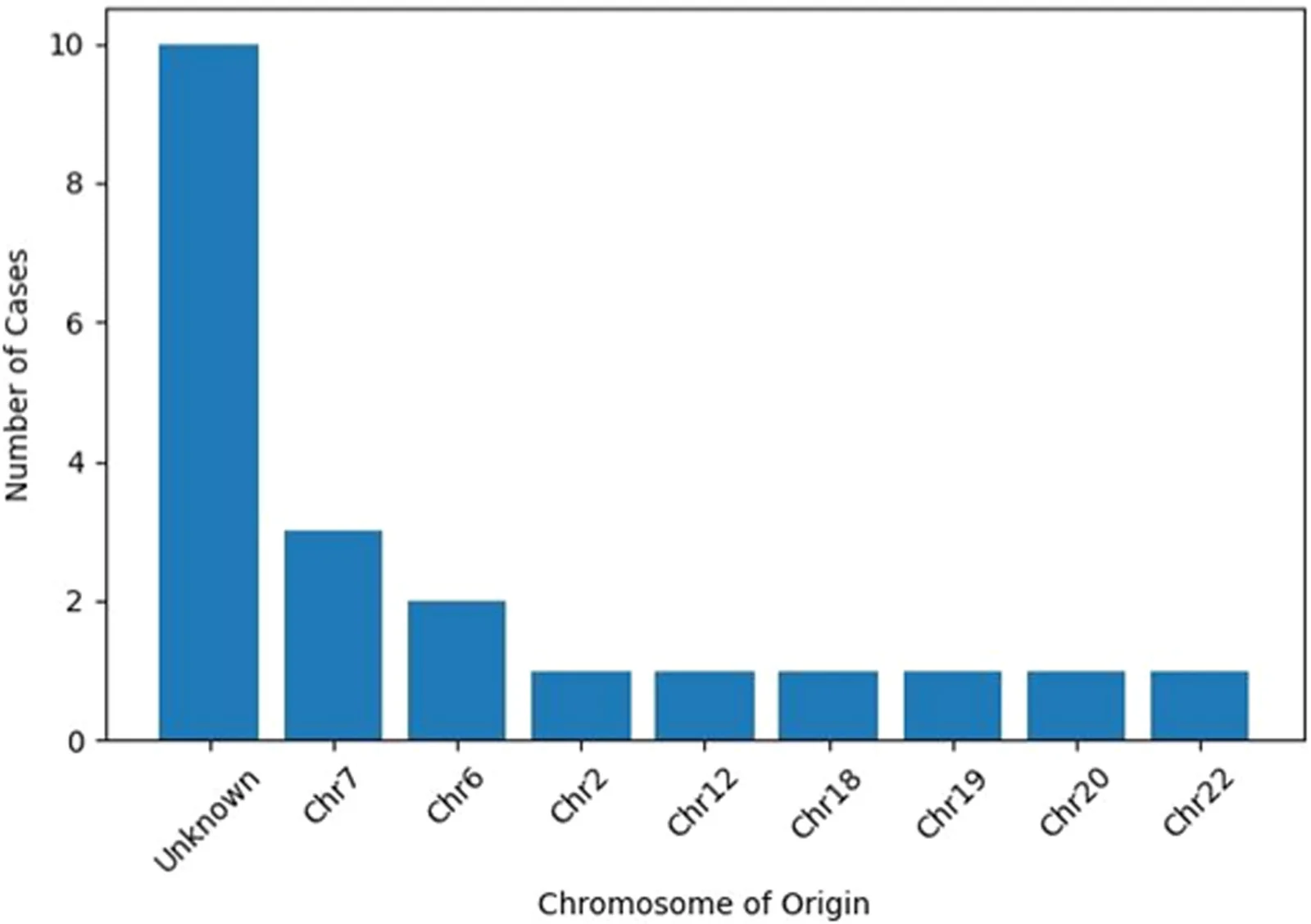
Origin of ring chromosomes.

Alterations involving chromosome 17p detected by conventional karyotyping were observed in 38.1% (n = 8) of the cases, while *TP53* deletions were identified by FISH in 19% (n = 4). *TP53* mutations were detected in 66.7% (n = 14) of patients, were absent in 19% (n = 4), and were indeterminate in 14.3% (n = 3). Considering *TP53* multi-hit status, 52.4% (n = 11) of patients were classified as multi-hit, 33.3% (n = 7) as non–multi-hit, and 14.3% (n = 3) as indeterminate. A strong association was observed between *TP53* multi-hit status and increasing karyotype complexity, particularly among patients with VCKs (Figure 2).

**Figure 2:**
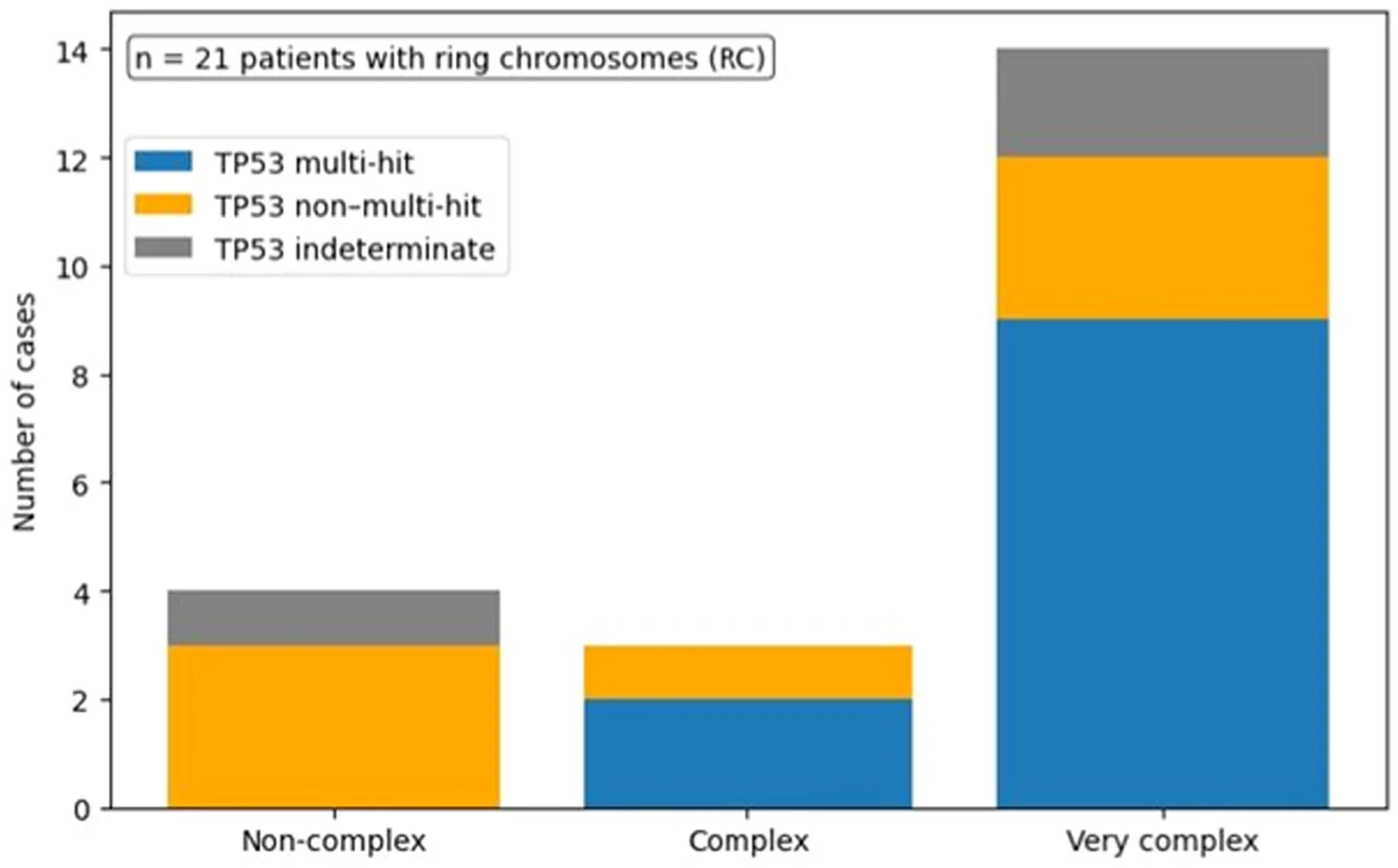
Relationship between ring chromosomes, karyotype complexity and *TP53* status.

During the follow-up, 19 patients (90.5%) died. Median overall survival (OS) was 3.2 months (mean = 6.8 months). In univariate survival analysis, *TP53* multi-hit status and the number of karyotypic abnormalities were significantly associated with increased mortality (Hazard ratio [HR]: 10.63; 95% CI: 2.25–50.31; p = 0.001 and HR: 1.06; 95% CI: 1.00–1.11; p = 0.043, respectively), as detailed in Table 2. Median OS for patients with non-complex karyotypes, CKs, and VCKs was 20.7, 0.5, and 1.2 months, respectively. Patients with VCKs had a 5.53-fold higher risk of death compared with those with non-complex karyotypes (95% CI: 1.12–27.28). Kaplan–Meier survival curves stratified by *TP53* multi-hit status are shown in Figure 3; these demonstrate markedly inferior survival among patients with *TP53* multi-hit alterations.

**Table 2:** Variables associated with overall survival.

**Variable**	**Estimated median time (months)**	**95% Confidence interval**		**Hazard ratio**	**95% Confidence interval**		**Deaths**	**Total N°**	**%**	**p-value**
		**Lower**	**Upper**		**Lower**	**Upper**				
**Age (years)**				1.01	0.96	1.06				0.713*
**Sex**										0.952
Female	1.7	0.0	5.2	1.00			6	7	85.7	
Male	3.2	0.0	8.8	1.03	0.38	2.76	13	14	92.9	
**Final Diagnosis**										0.482
Others	5.4	0.0	13.8	1.00			11	12	91.7	
AML	1.7	0.1	3.4	1.39	0.54	3.59	8	9	88.9	
***TP53* Deletion**										0.366
Negative	3.2	0.0	8.9	1.00			15	17	88.2	
Positive	0.4	0.0	4.5	1.65	0.53	5.14	4	4	100.0	
**N° of KT abn**				1.06	1.00	1.11				0.043*
**17p Alteration KT**										0.722
No	4.2	0.0	9.2	1.00			11	13	84.6	
Yes	1.0	0.0	2.9	1.18	0.46	3.06	8	8	100.0	
**Complexity**										**0.054**
Non-complex	20.7	0.0	43.5	1.00			2	4	50.0	
Complex	0.5	0.0	1.3	2.31	0.32	16.51	3	3	100.0	
Very complex	1.2	0.0	2.5	5.53	1.12	27.28	14	14	100.0	
***TP53* Mutation**										0.137
Absent	20.7	0.0	49.0	1.00			2	4	50.0	
Indeterminate	3.2	0.0	6.5	4.67	0.71	30.65	3	3	100.0	
Present	0.5	0.0	1.7	3.90	0.86	17.79	14	14	100.0	
**Multi-hit *TP53* Alteration**										**0.001**
Absent	20.7	5.0	36.4	1.00			5	7	71.4	
Indeterminate	3.2	0.0	6.5	7.84	1.19	51.68	3	3	100.0	
Present	0.4			10.63	2.25	50.31	11	11	100.0	
**General**	**3.2**	**0.0**	**7.7**				**19**	**21**	**90.5**	

Log-rank test; * Result from bivariate Cox regression.

KT: karyotype; Abn: abnormalities.

**Figure 3:**
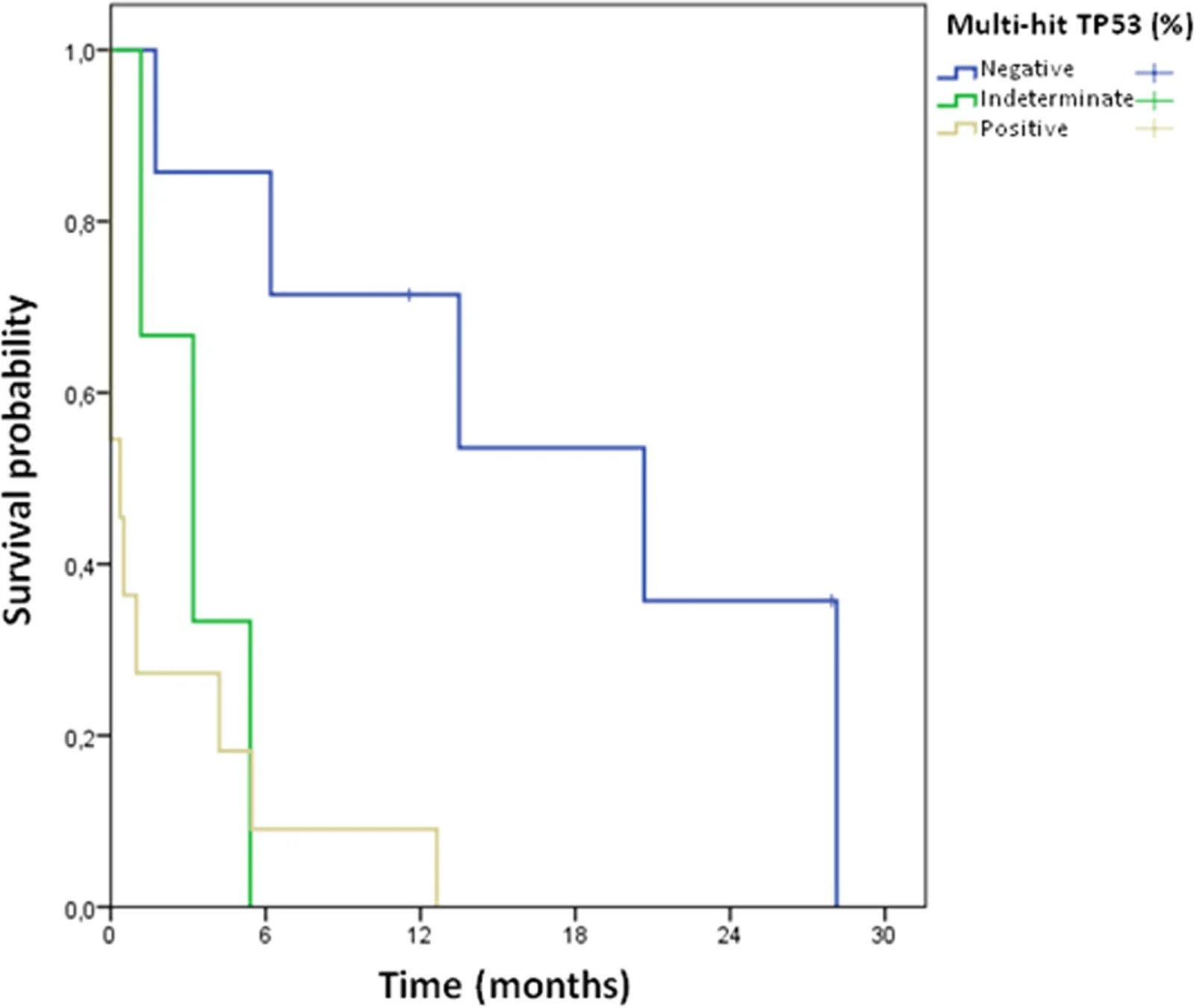
Survival probability of patients according to thier multi-hit *TP53* alteration.

## Discussion

According to Li et al. [1], current cytogenetic laboratories have an estimated detection rate of approximately 0.06% for constitutional RCs. There are no consistent data regarding the frequency of acquired RCs in human neoplasms. In this study, conducted between 2017 and 2022, RCs were detected in 0.29% of onco-hematological karyotypes, confirming that they represent a rare chromosomal aberration in hematological neoplasms. These cases occurred predominantly in older patients with a median age of 69 years. The most frequent diagnoses were AML and MDS, entities known to be associated with advanced age, cumulative genomic damage, and marked cytogenetic instability.

Several authors [2,5,12,13,23] have reported that karyotypes with three or more independent chromosomal abnormalities, confer a poor prognosis in myeloid neoplasms, particularly AML and MDS. Rosenbaum et al. [12] conducted the first large-scale systematic investigation (n = 96) addressing the clinical significance of RCs in AML and MDS, based on cytogenetic reports from patients treated at three major U.S. hospitals between 2004 and 2014. In that study, CKs or VCKs were identified in 75% of patients with acute myeloid leukemia and 97% of those with myelodysplastic syndromes. These findings indicate that ring chromosomes predominantly occur within highly complex cytogenetic profiles. Furthermore, MDS patients with both complex karyotypes (CKs or VCKs) and RCs had shorter leukemia-free survival (3.9 months) compared with those with RCs in the absence of CKs and VCKs (5.6 months). Among AML patients, the overall survival was similarly poor in those with CKs or VCKs regardless of the presence of RCs (4.8 months for patients with CKs or VCKs with RCs versus 5.3 months for those without RCs), whereas patients with RCs but without CKs and VCKs showed longer survival (19.8 months). These findings support the conclusion that karyotype complexity, rather than the presence of a RC itself is the major determinant of adverse prognosis, with RCs likely reflecting the underlying genomic instability of myeloid neoplasms with CKs or VCKs. The observation that RCs may disappear after therapy or emerge at relapse further supports their association with chromosomal instability rather than with disease initiation.

In the present cohort, patients with RCs presented a mean of 11 chromosomal abnormalities with 66.7% exhibiting VCKs. These findings demonstrated that an increasing number of chromosomal abnormalities was directly associated with reduced survival (HR: 1.06; 95% CI: 1.00–1.11; p = 0.043), and that patients with VCKs had a 5.53-fold higher risk of death (95% CI: 1.12–27.28) compared with those with non-complex karyotypes. Interestingly, median overall survival was lower in patients with complex karyotypes (0.5 months) than in those with very complex karyotypes (1.2 months). Although unexpected, this finding may reflect the limited cohort size and clinical heterogeneity of the cases analyzed. Moreover, 90.5% of patients died during follow-up, all of whom had CKs or VCKs. These findings are consistent with previous studies and reinforce the concept that RCs occur in the setting of advanced karyotypic evolution. Rather than acting as independent prognostic drivers, RCs appear to accompany severe genomic instability and do not substantially modify the already poor prognosis associated with CKs and VCKs.

Regarding the chromosomal origin of RC, a study of 125 AML cases with CKs or VCKs detected RCs in only 15 cases, and across published series, approximately 5.9% of MDS cases were reported to harbor RCs [2]. Rosenbaum et al. observed that, when identifiable, the most common RC was r(7), followed by r(6) [12]. Monosomy 7 or deletion of chromosome 7 are well-established abnormalities in MDS and AML and frequently involve telomeric regions, suggesting that r(7) may functionally resemble large 7q deletions. In a comprehensive review by Gebhart, chromosome 11 was the most frequently involved (approximately 15%), followed by chromosomes 7 (14%), 21 (9%), and 5 (6%), while other chromosomes appeared to be affected more randomly [2]. Li et al. reported r(21) in association with AML [1]. In the current cohort, RCs were derived predominantly from chromosomes 6 and 7. In partial agreement with previous studies, the predominance of r(7) is consistent with the high frequency of involvement of chromosome 7 in myeloid neoplasms characterized by CKs or VCKs and marked genomic instability. The recurrent involvement of chromosome 6 may similarly reflect its susceptibility to large structural rearrangements in advanced myeloid disease. The absence of r(11) and r(21) in this cohort, as well as the sporadic occurrence of RCs derived from other chromosomes (2, 12, 18, 19, 20, and 22), likely reflects the sample size, disease heterogeneity, and variability in cytogenetic landscapes across studies.

Alterations of the tumor suppressor gene *TP53*, including deletions and mutations, have been widely described as central events in the pathogenesis and progression of hematological malignancies [5,18,19,24,25]. *TP53* dysfunction compromises DNA damage response, cell cycle control, and chromosomal segregation, thereby promoting chromosomal breakage and genomic instability. In this study, *TP53* deletion was identified in approximately 19% of cases, while *TP53* mutations were detected in 66.7% of patients, with 38.1% showing 17p alterations by conventional cytogenetics. Importantly, *TP53* multi-hit alterations were significantly associated with increased mortality (HR: 10.63; 95% CI: 2.25–50.31; p = 0.001), as was the increasing number of chromosomal abnormalities (HR: 1.06; 95% CI: 1.00–1.11; p = 0.043). Although these findings are well established as adverse prognostic markers in myeloid neoplasms, their relevance in the present study lies in their indirect association with the presence of RCs. *TP53* dysfunction creates a permissive genomic environment for the accumulation of complex structural rearrangements and the formation of unstable chromosomal configurations, such as RCs. In this context, RCs do not appear to function as independent prognostic drivers, but rather as cytogenetic manifestations of profound genomic instability, frequently arising in association with *TP53* multi-hit alterations and VCKs.

Despite the observed association between RCs, karyotypic complexity, and *TP53* alterations, the results do not allow definitive conclusions regarding the temporal origin of RCs, and this hypothesis remains speculative. In this context, RCs may represent cytogenetic manifestations of extreme genomic instability associated with *TP53* dysfunction rather than independent leukemogenic events. Consistently, all patients who died in this cohort harbored at least one major adverse feature (*TP53* alteration, complex karyotype both CKs and VCKs, or both) whereas the only surviving patients had non-complex karyotypes and no *TP53* deletion or mutation. Therefore, while our data do not allow definitive conclusions regarding the temporal origin of RCs, they support the interpretation that RCs occur in highly unstable genomic backgrounds and are closely linked to *TP53*-driven clonal evolution.

## Conclusion

RCs were infrequent in myeloid neoplasms; however, when present, they were consistently associated with *TP53* alterations and increased karyotype complexity. In this cohort, *TP53* deletions and mutations and karyotype complexity emerged as independent prognostic risk factors, each strongly associated with an extremely poor outcome. All patients harboring *TP53* alterations and all those with CKs or VCKs died, whereas survival was observed only in patients without *TP53* alterations and with non-complex karyotypes. These findings highlight the central prognostic relevance of *TP53* status and karyotypic complexity in myeloid neoplasms with RCs, reinforcing their role as key determinants of disease aggressiveness and clinical outcome.

## Conflicts of interest

None.

## Data Availability

The data that support the findings of this study are available from the corresponding author upon reasonable request.

## References

[1] Li P., Dupont B., Hu Q., Crimi M., Shen Y., Lebedev I. (2022). The past. present. and future for constitutional ring chromosomes: a report of the international consortium for human ring chromosomes. HGG Adv.

[2] Gebhart E. (2008). Ring chromosomes in human neoplasias. Cytogenet Genome Res.

[3] Pristyazhnyuk I.E., Menzorov A.G. (2018). Ring chromosomes: from formation to clinical potential. Protoplasma.

[4] Zeng T., Huang W., Cui L., Zhu P., Lin Q., Zhang W. (2022). The landscape of extrachromosomal circular DNA (eccDNA) in the normal hematopoiesis and leukemia evolution. Cell Death Discov.

[5] Shahjahani M., Hadad E.H., Azizidoost S., Nezhad K.C., Shahrabi S. (2019). Complex karyotype in myelodysplastic syndromes: diagnostic procedure and prognostic susceptibility. Oncol Rev.

[6] McGowan-Jordan J, Hastings RJ, M. S. ISCN 2020: an International System for Human Cytogenomic Nomenclature (2020) reprint of 'cytogenetic and genome research 2020;160(7–8). doi: 10.1159/isbn.978-3-318-06867-2. 10.1159/000516655 34407535

[7] Guilherme R.S., Meloni V.F.A., Kim C.A., Pellegrino R., Takeno S.S., Spinner N.B. (2011). Mechanisms of ring chromosome formation, ring instability and clinical consequences. BMC Med Genet.

[8] Yip M.Y. (2015). Autosomal ring chromosomes in human genetic disorders. Transl Pediatr.

[9] Marques F.K., Marinho F.L.O., Araújo S.S.S., Sabino A.P. (2021). Investigação de mutações em neoplasias mieloides utilizando um painel multigene de sequenciamento de nova geração. Hematol Transfus Cell Ther.

[10] Lopes L.P., Santos C.S., Severino G.S., Freitas T.R., Caetano I.M., Varotti F.P. (2022). Abordagens do tratamento da leucemia mieloide aguda: revisão integrativa. BJD.

[11] Silva E.B., Nascimento J.A. (2018). O diagnóstico das síndromes mielodisplásicas: revisão da literatura. RBAC.

[12] Rosenbaum M.W., Pozdnyakova O., Geyer J.T., Cin P.D., Hasserjian R.P. (2017). Ring chromosome in myeloid neoplasms is associated with complex karyotype and disease progression. Hum Pathol.

[13] Ornellas M.H.F., Maioli M.C.P., Lucena S.B.S.G., Bastos E.F., Chaves T.F., de Melo K.V. (2018). Complex karyotype including ring chromosome 11 in a patient with acute myeloid leukemia: case report. Sao Paulo Med J.

[14] Chrzanowska N.M., Kowalewski J., Lewandowska M.A. (2020). Use of fluorescence In situ hybridization (FISH) in diagnosis and tailored therapies in solid tumors. Molecules.

[15] Ankathil R., Ismail S.M., Yunus N.M., Sulong S., Husin A., Abdullah A.D. (2020). Clinical implications of conventional cytogenetics, fluorescence in situ hybridization (FISH) and molecular testing in chronic myeloid leukaemia patients in the tyrosine kinase inhibitor era – a review. Malays J Pathol.

[16] Aikawa V.N., Leal A.M., Kishimoto R.K., Velloso E.D.R.P. (2016). Citogenética nas Neoplasias Hematológicas. Clínica Médica.

[17] GeneCards – the human gene database [homepage na internet]. TP53 Gene - Tumor Protein P53 [accessed 21 August 2023]. Available at: ​https://www.genecards.org

[18] Chung J., Sallman D.A., Padron E. (2019). TP53 and therapy-related myeloid neoplasms. Best Pr Res Clin Haematol.

[19] Prokocimer M., Molchadsky A., Rotter V. (2017). Dysfunctional diversity of p53 proteins in adult acute myeloid leukemia: projections on diagnostic workup and therapy. Blood.

[20] Gohring G., Michalova K., Beverloo H.B., Betts D., Harbott J., Haas O.A. (2010). Complex karyotype newly defined: the strongest prognostic factor in advanced childhood myelodysplastic syndrome. Blood.

[21] Khoury J.D., Solary E., Abla O., Akkari Y., Alaggio R., Apperley J.F. (2022). The 5th edition of the world health organization classification of haematolymphoid tumours: myeloid and histiocytic/dendritic neoplasms. Leukemia.

[22] Arber D.A., Orazi A., Hasserjian R.P., Borowitz M.J., Calvo K.R., Kvasnicka H.M. (2022). International consensus classification of myeloid neoplasms and acute leukemias: integrating morphologic. clinical. and genomic data. Blood.

[23] Haase D., Germing U., Schanz J., Pfeilstöcker M., Nösslinger T., Hildebrandt B. (2007). New insights into the prognostic impact of the karyotype in MDS and correlation with subtypes: evidence from a core dataset of 2124 patients. Blood.

[24] Rucker F.G., Schlenk R.F., Bullinger L., Kayser S., Teleanu V., Kett H. (2012). TP53 alterations in acute myeloid leukemia with complex karyotype correlate with specific copy number alterations. Monosomal karyotype. And dismal outcome. Blood.

[25] Wang W., Routbort M.J., Tang Z., Ok C.Y., Patel K.P., Daver N. (2017). Characterization of TP53 mutations in low-grade myelodysplastic syndromes and myelodysplastic syndromes with a non-complex karyotype. Eur J Haematol.

